# Safety and antitumor activity of the anti–PD-1 antibody pembrolizumab in patients with advanced, PD-L1–positive papillary or follicular thyroid cancer

**DOI:** 10.1186/s12885-019-5380-3

**Published:** 2019-03-04

**Authors:** Janice M. Mehnert, Andrea Varga, Marcia S. Brose, Rahul R. Aggarwal, Chia-Chi Lin, Amy Prawira, Filippo de Braud, Kenji Tamura, Toshihiko Doi, Sarina A. Piha-Paul, Jill Gilbert, Sanatan Saraf, Pradeep Thanigaimani, Jonathan D. Cheng, Bhumsuk Keam

**Affiliations:** 1 Rutgers Cancer Institute of New Jersey, 195 Little Albany Street, New Brunswick, NJ 08901 USA; 2 Institut Gustave Roussy, 114, rue Edouard-Vaillant, 94800 Villejuif, France; 3 Department of Otorhinolaryngology, Head and Neck Surgery and the Abramson Cancer Center at the University of Pennsylvania, 3400 Spruce Street, Philadelphia, PA 19104 USA; 4 University of California, San Francisco Cancer Center, 500 Parnassus Avenue, San Francisco, CA 94143 USA; 5 Department of Oncology, National Taiwan University Hospital, 7 Chung-Shan S Rd, Taipei, 10002 Taiwan; 6 Division of Medical Oncology and Hematology, Princess Margaret Cancer Centre, 610 University Avenue, Toronto, ON M5G 2C1 Canada; 7 Department of Oncology, University of Milan and Fondazione IRCCS Istituto Nazionale Tumori Milano, Via Giacomo Venezian 1, 20133 Milan, Italy; 8 Department of Breast and Medical Oncology, National Cancer Center Hospital Tokyo, 5-1-1 Tsukiji, Chuo-ku, Tokyo, 104-0045 Japan; 9 Department of Gastrointestinal Oncology, National Cancer Center Hospital East, 6-5-1 Kashiwanoha, Kashiwa, Chiba, 277-8577 Japan; 10 Department of Investigational Cancer Therapeutics, The University of Texas MD Anderson Cancer Center, 1515 Holcombe Boulevard, Houston, TX 77030 USA; 11 Hematology/Oncology, Vanderbilt University School of Medicine, 215 Light Hall, Nashville, TN 37232 USA; 12 Merck & Co., Inc., 8000 Galloping Hill Road, Kenilworth, NJ 07033 USA; 13 Department of Internal Medicine, Seoul National University Hospital, 101, Daehak-Ro, Jongno-Gu, Seoul, 03080 Republic of Korea

**Keywords:** Thyroid cancer, Immunotherapy, Pembrolizumab, Anti–PD-1, PD-1, PD-L1

## Abstract

**Background:**

Treatment options for advanced thyroid cancer refractory to standard therapies are limited. The safety and efficacy of pembrolizumab were evaluated in patients with advanced differentiated thyroid cancer expressing programmed death ligand 1 (PD-L1).

**Methods:**

Patients with advanced thyroid cancer were enrolled in the nonrandomized, phase Ib KEYNOTE-028 trial conducted to evaluate safety and antitumor activity of the anti–programmed death 1 (PD-1) antibody pembrolizumab in advanced solid tumors. Key eligibility criteria were advanced papillary or follicular thyroid cancer, failure of standard therapy, and PD-L1 expression in tumor or stroma cells (assessed by immunohistochemistry). Pembrolizumab 10 mg/kg was administered every 2 weeks up to 24 months or until confirmed progression or intolerable toxicity. The primary endpoint was objective response rate (ORR) per Response Evaluation Criteria in Solid Tumors, version 1.1.

**Results:**

Twenty-two patients were enrolled: median age was 61 years; 59% were women; and 68% had papillary carcinoma. Median follow-up was 31 months (range, 7–34 months). Treatment-related adverse events were observed in 18 (82%) patients; those occurring in ≥15% of patients were diarrhea (*n* = 7) and fatigue (*n* = 4). One grade ≥ 3 treatment-related adverse event occurred (colitis, grade 3); no treatment-related discontinuations or deaths occurred. Two patients had confirmed partial response, for an ORR of 9% (95% confidence interval [CI], 1–29%); response duration was 8 and 20 months. Median progression-free survival was 7 months (95% CI, 2–14 months); median overall survival was not reached (95% CI, 22 months to not reached).

**Conclusions:**

Results of this phase Ib proof-of-concept study suggest that pembrolizumab has a manageable safety profile and demonstrate evidence of antitumor activity in advanced differentiated thyroid cancer in a minority of patients treated. Further analyses are necessary to confirm these findings.

**Trial registration:**

Clinicaltrials.gov identifier: NCT02054806. Registered 4 February 2014.

**Electronic supplementary material:**

The online version of this article (10.1186/s12885-019-5380-3) contains supplementary material, which is available to authorized users.

## Background

Thyroid cancer is the most common endocrine malignancy and the eighth most common cancer in the United States [1, 2]. Although the prognosis for most thyroid cancers is generally good (98% overall 5-year survival rate) [2], approximately 10% of patients with differentiated thyroid cancer develop progressive invasive primary disease, 5% develop distant metastases, and 20–30% experience disease recurrence [3].

Metastatic disease is usually treated with a combination of surgery and radioiodine ablation (RAI), and success depends on whether metastasis is in a location amenable to surgical resection or radioiodine uptake in the tumor tissue is significant [1, 4]. Since the identification of multiple kinase inhibitors (MKIs), more treatment options for recurrent/metastatic thyroid cancer are available [5]. Two MKIs—sorafenib [6, 7] and lenvatinib [8, 9]—have been approved in many countries for treatment of advanced differentiated thyroid cancer. Results of a phase III randomized trial revealed a progression-free survival (PFS) benefit with sorafenib compared with placebo in patients with RAI-refractory, advanced, differentiated thyroid cancer [10]. Additionally, lenvatinib was also associated with significant improvement in PFS and objective response rate (ORR) compared with placebo; median overall survival (OS) was not reached after a median follow-up of 17 months. Lenvatinib was associated with considerable treatment-related toxicity, with a drug-related mortality rate of 2% [11]. Sorafenib and lenvatinib are now recommended by the National Comprehensive Cancer Network for the treatment of progressive, RAI-refractory differentiated thyroid carcinoma [4]. However, despite being effective, their duration of response is limited, and disease ultimately progresses. Therefore, new therapies are needed.

The development and approval of immunotherapeutics for cancer, and immune checkpoint inhibitors such as anti–CTLA-4 and anti–programmed death 1 (PD-1) agents in particular, have altered the treatment landscape for many malignancies [12]. They act to re-establish immune surveillance, from which some cancers are able to hide [12, 13]. The clinical benefit observed with immune checkpoint inhibitors varies with both the immunotherapeutic agent and the type of cancer. However, PD-1 blockade appears to be effective for a wide variety of tumor types [12]. Thyroid cancer cells are known to produce cytokines and chemokines that are able to promote tumorigenesis. In aggressive, recurrent papillary thyroid cancer, the frequency of regulatory T cells is increased, and expression of PD-1 ligand 1 (PD-L1) is correlated with greater risk of recurrence and reduced disease-free survival [12]. Targeting of these immune system components may thus prove useful in the treatment of thyroid cancer. The application of immune checkpoint inhibitors in advanced thyroid cancer has not been well studied to date.

The PD-1 immune checkpoint pathway regulates induction and maintenance of peripheral immune tolerance via engagement between the PD-1 receptor (expressed on monocytes and T, B, and natural killer cells) and its ligands PD-L1 and PD-L2 [14]. Upregulation of this pathway induces suppression of immune response in many tumors, allowing them to escape immune surveillance [15, 16]. Expression of PD-L1 in advanced differentiated thyroid carcinomas, when detected, has been associated with aggressive disease and poor prognosis, making anti–PD-1 therapy a potential treatment option [3, 17, 18].

Pembrolizumab is a fully humanized, selective immunoglobulin G4/κ anti–PD-1 monoclonal antibody that exhibits antitumor activity by blocking interaction between PD-1 and its ligands. It has been found to be effective in head and neck cancers [19–21]. Pembrolizumab has demonstrated robust antitumor activity and a favorable safety profile in multiple tumor types, and it is currently approved in more than 60 countries for one or more advanced malignancies.

The aim of this study was to assess the safety, tolerability, and antitumor activity of pembrolizumab in patients with PD-L1–positive, advanced thyroid cancer who were enrolled in the phase Ib KEYNOTE-028 trial.

## Methods

### Study design and patients

Eligibility criteria were age ≥ 18 years; presence of cytologically or histologically confirmed, PD-L1–positive, locally advanced or metastatic follicular or papillary thyroid cancer in which, per the opinion of the treating physician, previous standard therapy was ineffective, did not occur, or was not considered appropriate; measurable disease based on Response Evaluation Criteria In Solid Tumors, version 1.1 (RECIST v1.1); Eastern Cooperative Oncology Group (ECOG) performance status 0 or 1; and adequate organ function. Patients were not required to be radioactive iodine refractory in order to be enrolled in the study. Exclusion criteria were current or past participation in a study of an investigational agent or investigational device ≤4 weeks before the first dose of treatment; prior anticancer monoclonal antibody therapy ≤4 weeks before the first pembrolizumab dose; immunosuppressive therapy or diagnosis of immunodeficiency therapy ≤7 days before the first pembrolizumab dose; chemotherapy, targeted small-molecule therapy, or radiation therapy ≤2 weeks before the first pembrolizumab dose; therapy with any anti–PD-1, anti–PD-L1, or immune checkpoint inhibitor; active autoimmune disease that necessitated systemic treatment in the preceding 2 years; interstitial lung disease; known additional malignancy that was progressing or necessitated treatment; and active brain metastases. The study protocol and all amendments were approved by the institutional review board or ethics committee of each participating site and were conducted per the principles expressed in the Declaration of Helsinki. All patients provided written informed consent to participate. This trial was registered with Clinicaltrials.gov, identifier NCT02054806.

### Treatment and assessments

Pembrolizumab 10 mg/kg was administered every 2 weeks via 30-min infusion. Treatment continued for 24 months or until confirmed progressive disease, unacceptable adverse events (AEs), or investigator or patient decision to withdraw. Dosing was interrupted because of unacceptable toxicity but could resume after resolution of toxicity to grade 0/1 within 12 weeks of the last infusion. Response was assessed by computed tomography or magnetic resonance imaging every 8 weeks for the first 6 months and every 12 weeks thereafter. Patients who discontinued pembrolizumab after substantiated complete response (CR) after ≥24 weeks of therapy and ≥ 2 treatments after initial CR, or who discontinued after receiving pembrolizumab for ≥24 months for reasons other than progressive disease or unacceptable toxicity could be eligible for up to 1 year of re-treatment after substantiation by radiographic progressive disease. Patients were permitted to continue to treatment after the onset of progressive disease if the patient’s condition was clinically stable in the investigator’s judgment. AEs were graded per the National Cancer Institute Common Terminology Criteria for Adverse Events, version 4.0, and were monitored throughout the study and for 30 days after treatment discontinuation (90 days for serious AEs). Immune-mediated AEs were also reported and were defined as events with potentially drug-related immunologic causes that were consistent with an immune phenomenon, regardless of whether they were attributable to the study drug or to an immune response.

Tumor PD-L1 status was determined at a central laboratory during the screening period using either an archived formalin-fixed, paraffin-embedded tumor sample or a newly obtained biopsy sample. PD-L1 expression was assessed using a prototype immunohistochemistry assay (QualTek Molecular Laboratories, Goleta, California) [22] and the 22C3 antibody (Merck & Co., Inc., Kenilworth, New Jersey). PD-L1 positivity was defined as membranous staining on ≥1% modified proportion score or interface pattern as described previously [22]. Note that tumor markers and laboratory evaluations specific for thyroid cancer were not required to be captured by investigators because of the signal-finding nature of the study.

### Endpoints

Primary endpoints were ORR, defined as the proportion of patients with a best overall response of confirmed CR or partial response (PR) per RECIST v1.1 by investigator review, and safety and tolerability. Secondary endpoints were PFS (time from enrollment to the first documented occurrence of PD per RECIST v1.1 or death from any cause, whichever occurred first), OS (time from enrollment to death from any cause), and duration of response (time from first RECIST v1.1–based response to progressive disease in patients who experience PR or better).

### Statistical analyses

The binomial exact method was used for power and sample size calculations. A sample size of 22 evaluable patients in this cohort was calculated to provide 80% power to demonstrate that the best ORR exceeded 10% at an overall one-sided 8% α level if the true best ORR was 35%. The efficacy population was composed of all patients who received ≥1 dose of pembrolizumab and had measurable disease at baseline per RECIST v1.1. The safety population was composed of all patients who received ≥1 dose of pembrolizumab. A truncated sequential probability test was used to evaluate ORR; PFS, OS, and duration of response were estimated using the Kaplan-Meier method. The data cutoff was February 20, 2017.

## Results

### Patient baseline characteristics and treatment

Fifty-one patients with thyroid cancer were screened; of 36 (71%) with PD-L1–positive tumors, 22 were enrolled (per eligibility criteria) and received ≥1 dose of pembrolizumab. Baseline characteristics are listed in Table 1; median age was 61 years (range, 23–76 years); 13 (59%) were women; and 12 (55%) had an Eastern Cooperative Oncology Group (ECOG) performance status of 0. Relative proportions of patients with papillary and follicular thyroid cancer were approximately 2:1 (papillary, 68%; follicular, 32%). Nine (41%) patients had ≥2 prior lines of therapy for advanced disease; 11 (50%) patients had previously received an MKI. The most frequent prior treatment was RAI (18 patients [82%]), followed by sorafenib (7 [32%]) and pazopanib (3 [14%]). Among the 22 PD-L1–positive patients enrolled in this cohort, 20 (91%) were PD-L1 positive in the tumor only and 2 (9%) were positive in the tumor and the stroma.

**Table 1:** Baseline demographics and clinical characteristics

Characteristic, n (%)^a^	*N* = 22
Median age, years (range)	61 (23–76)
Sex	
Male	9 (41)
Female	13 (59)
Race	
Asian	7 (32)
Multiracial	1 (5)
White	14 (64)
ECOG PS	
0	12 (55)
1	10 (45)
Thyroid cancer histology	
Papillary	15 (68)
Follicular	7 (32)
Treatment-naive^b^	
Yes	6 (27)
No	16 (73)
Prior lines of therapy for advanced disease	
1	7 (32)
2	5 (23)
3	3 (14)
4	1 (5)
Unknown	6 (27)
Select prior therapies^c^	
Iodine	18 (82)
Sorafenib	7 (32)
Pazopanib	3 (14)
Lenalidomide	2 (9)
Cediranib	2 (9)
Vemurafenib	2 (9)
Sunitinib	2 (9)
Everolimus	2 (9)
Paclitaxel	2 (9)

*ECOG PS* Eastern Cooperative Oncology Group performance status

^a^Totals may equal > 100% because of rounding

^b^Patients had received no prior oncologic or biologic drugs but may have received iodine radiotherapy or surgery

^c^Patients may have received more than 1 prior treatment listed

Median follow-up was 31 months (range, 7–34 months). At the data cutoff date, 18 patients (82%) had discontinued the study: 10 because of PD, 7 because of patient or physician decision, and 1 was lost to follow-up; 4 remained on study.

### Safety

Eighteen patients (82%) experienced treatment-related AEs, most commonly diarrhea (7 [32%]), fatigue (4 [18%]), pruritus (3 [14%]), and rash (3 [14%]); all but 1 were grade 1 or 2 (Table 2). No grade 4 treatment-related AEs or treatment-related deaths or discontinuations occurred. Immune-mediated AEs were reported in 5 patients: pneumonitis (2 patients, 1 each of grades 1 and 2), interstitial lung disease (1 patient, grade 1), colitis (1 patient, grade 3), and hypothyroidism (1 patient, grade 2).

**Table 2:** Treatment-related adverse events: all grades occurring in ≥2 patients and grade 3-5^a^ occurring in any patient

Treatment-related adverse events (*N* = 22)	All grades *n* (%)	Grade 3-5^a^ *n* (%)
All	18 (82)	1 (5)
Diarrhea	7 (32)	0
Fatigue	4 (18)	0
Pruritus	3 (14)	0
Rash	3 (14)	0
Decreased appetite	2 (9)	0
Headache	2 (9)	0
Cough	2 (9)	0
Pneumonitis	2 (9)	0
Colitis	1 (5)	1 (5)

^a^There were no grade 4 or 5 treatment-related adverse events

### Antitumor activity

**Fig. 1:**
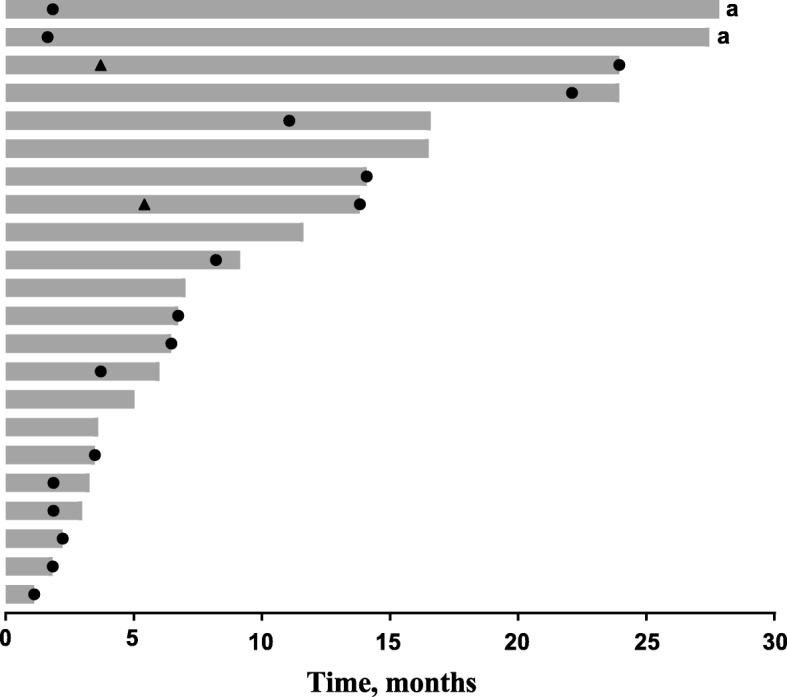
Duration of exposure to pembrolizumab and summary of best overall response (*N* = 22) ^a^Patient was considered clinically stable per investigator’s judgment and was permitted to continue treatment after progressive disease

**Fig. 2:**
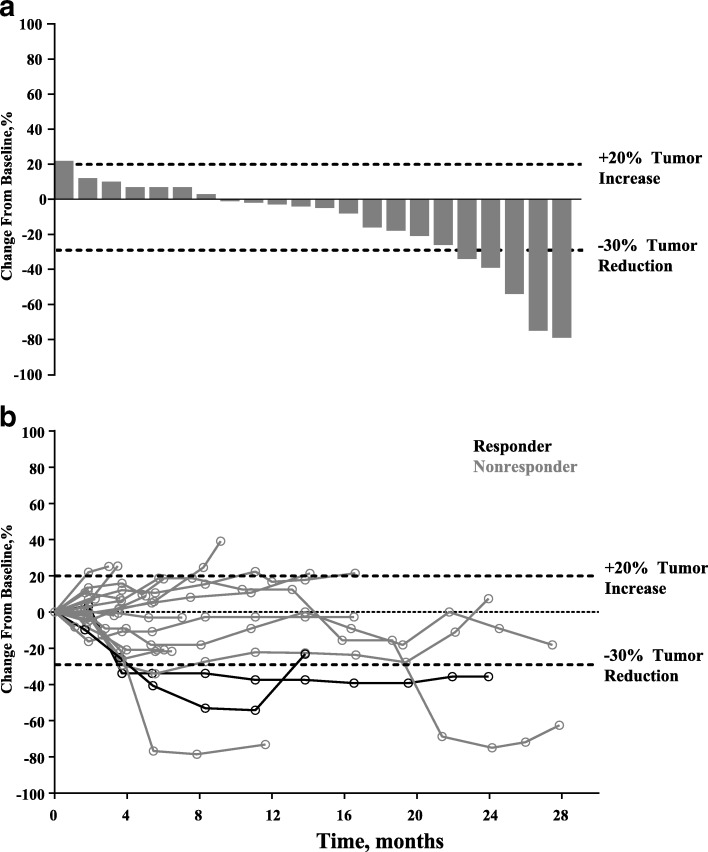
**a** Change from baseline in sum of longest diameters of target lesions (*n* = 21) and (**b**) change from baseline over time (*n* = 21)

Confirmed PR (by investigator review) was observed in 2 patients, for an ORR of 9% (95% confidence interval [CI], 1–29%) (Table 3). Time to response for the 2 patients with substantiated PR were 4 and 5 months, respectively, with response durations of 20 and 8 months, respectively (Fig. 1). Both patients had papillary thyroid cancer. Stable disease (SD) was experienced by 13 patients (59%; 95% CI, 36–79%), with a median duration of 7 months yielding a disease control rate (i.e. confirmed PR + SD) of 68%. SD was achieved by 57% (4/7) of patients with follicular histology and 60% (9/15) of patients with papillary histology. The clinical benefit rate (i.e. confirmed PR + SD ≥6 months) was 50% (95% CI, 28–72%). Seven patients had progressive disease as best response (32%; 95% CI, 14–55%). Of these, 3 patients had follicular cancer and 4 patients had papillary thyroid cancer, representing 43 and 27% of the respective histologic subgroups. Reduction in tumor size (sum of the longest diameter) from baseline was observed in 15 (68%) of 22 patients in whom this parameter was evaluable and was generally maintained (Fig. 2).

**Table 3:** Antitumor activity

Response evaluation (*N* = 22)	*n*	% (95% CI) or median (range)
ORR^a,b^, % (95% CI)	2	9 (1–29)
CR	0	0 (0–15)
PR	2	9 (1–29)
SD	13	59 (36–79)
PD	7	32 (14–55)
CBR^b^, % (95% CI)	11	50 (28–72)
SD ≥6 months, % (95% CI)	9	69 (39–91)
TTR (months), median (range)	2	5 (4–5)
DOR (months), median (range)	2	14 (8–20)
Follow-up duration (months), median (range)	22	31 (7–34)

Abbreviations: *CBR* clinical benefit rate, *CR* complete response, *DOR* duration of response; *NR* not reached, *ORR* objective response rate, *PD* progressive disease, *PR* partial response, *SD* stable disease, *TTR* time to response

^a^ORR = CR + PR

^b^Confirmed by investigator review

^c^CBR = CR + PR + SD ≥6 months

**Fig. 3:**
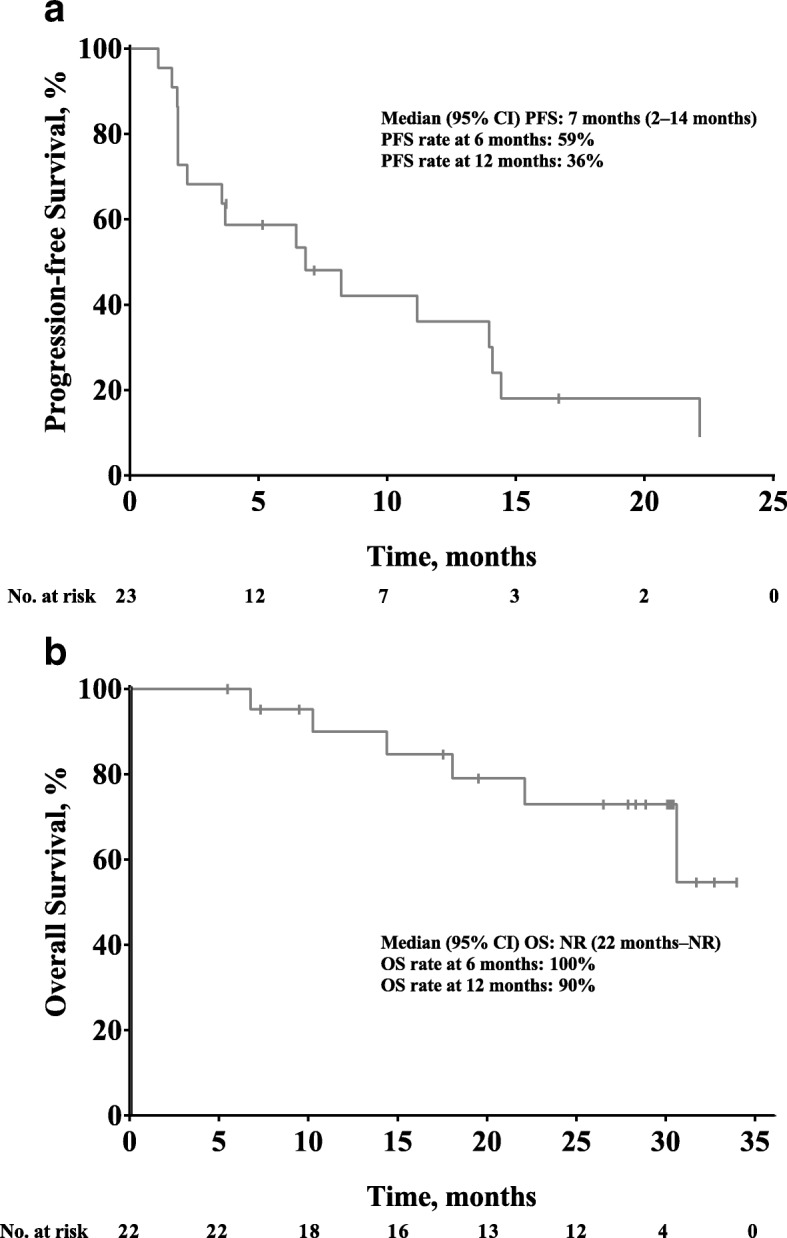
Kaplan-Meier estimates (*N* = 22) of (**a**) PFS and (**b**) OS

Median PFS was 7 months (95% CI, 2–14 months), and 6- and 12-month PFS rates were 59 and 36%, respectively. Median OS was not reached (95% CI, 22 months to not reached), with 6- and 12-month OS rates of 100 and 90%, respectively (Fig. 3).

## Discussion

Until recently, treatment options for advanced differentiated thyroid carcinomas were limited to surgery and RAI [4]. The recent approval of MKIs has improved the therapeutic arsenal, benefiting those whose tumors progress after RAI or for whom surgery is contraindicated. Nonetheless, disease in patients treated with approved agents will inevitably progress. Because thyroid cancer is a relatively common disease with a high unmet medical need in refractory patients, pembrolizumab was evaluated in a thyroid cancer cohort of KEYNOTE-028. In this phase Ib, proof-of-concept study, pembrolizumab was well tolerated in patients with advanced papillary or follicular thyroid cancer that had progressed with standard therapy, and no treatment-related discontinuations or deaths occurred. The safety profile was generally consistent with that observed previously for pembrolizumab [19, 20]. After a median follow-up of 31 months, confirmed ORR was 9%, disease control rate was 68%, and clinical benefit rate was 50%. Two patients had confirmed PR, and, in 13 other patients, median duration of SD was 7 months. Median PFS was 7 months and, although median OS was not reached at the data cutoff date, 6- and 12-month OS rates were high at 100 and 90%, respectively. Although PFS data in this study were greater than those of placebo-treated patients with RAI-refractory, differentiated thyroid cancer whose disease progressed after previous treatment (4–6 months) [10, 11], patients in KEYNOTE-028 were not required to have experienced disease progression with their previous treatment before study entry. These results must also be interpreted considering the biological behavior of advanced thyroid cancer, which is known to often have an indolent course. In addition, patients in KEYNOTE-028 were heavily pretreated; therefore, it is unclear what the PFS for this group would have been without treatment or how the effects of multiple prior therapies may confound these results. Nonetheless, objective responses were observed in a minority of patients treated. Hence, the observed response rates and PFS differences must be substantiated in subsequent clinical trials.

PD-L1 expression has been observed in differentiated thyroid tumors, including the papillary subtype. In one study, 59% (13/22) of differentiated thyroid tumors expressed PD-L1, and 50% of tumors contained PD-1–positive lymphocytes [18]. In another retrospective analysis, membranous PD-L1 expression was observed in 40% (74/185) of all surgically resected papillary thyroid tumors analyzed and in > 70% (53/74) of advanced-stage (III/IV) tumors [3]. In that study, the presence of PD-L1 staining was associated with significantly reduced disease-free survival [3]. The high membranous PD-L1 expression found in advanced-stage tumors suggests that, as in other PD-L1–expressing tumors, pembrolizumab could be an effective therapy for papillary and follicular thyroid cancer. However, archival tissue was permitted in this study and, in some cases, was taken from metastatic sites. In thyroid cancer, this can be a significant limitation because tumor dedifferentiation with progression is a frequent feature [23]. Therefore, the treated lesions may not represent differentiated thyroid carcinoma. Future investigation that requires fresh biopsy specimens taken immediately before therapy, when clinically feasible, may be informative.

Given that KEYNOTE-028 was a signal-finding study, two additional limitations, among others, that might have affected the generalizability of the results were that patients were not required to be iodine refractory before enrollment and that tumor marker and laboratory evaluations were not required to be collected, despite the fact that these items are specific to thyroid cancer. Additional studies that include these items will be needed to further confirm findings.

## Conclusion

Results of this proof-of-concept study suggest that pembrolizumab may be effective and have a favorable safety profile in PD-L1–positive thyroid cancer. These data may lay the foundation for further clinical evaluation of pembrolizumab to establish its place in the differentiated thyroid carcinoma therapeutic arena. Clinical benefit of a fixed dose of pembrolizumab (200 mg once every 3 weeks) in advanced differentiated thyroid cancer will be further investigated in the multicohort phase 2 KEYNOTE-158 trial (Clinicaltrials.gov identifier: NCT02628067).

## Additional file

Additional file 1: **Table S1.** Institutional Review Board or Ethics Committee of Each Participating Site. Description of data: Provides details of institutional review board/ethics committees for each participating site. (PDF 118 kb)

## Abbreviations

AE: Adverse event
CI: Confidence interval
CR: Complete response
ECOG: Eastern Cooperative Oncology Group
MKI: Multiple kinase inhibitor
ORR: Objective response rate
OS: Overall survival
PD-1: Programmed death 1
PD-L1: Programmed death ligand 1
PFS: Progression-free survival
PR: Partial response
RAI: Radioiodine ablation
RECIST v1.1: Response Evaluation Criteria In Solid Tumors, version 1.1
SD: Stable disease
