# Supplementary material for: Safety and antitumor activity of the anti–PD-1 antibody pembrolizumab in patients with advanced, PD-L1–positive papillary or follicular thyroid cancer
Source: BMC Cancer. 2019 Mar 4;19:196. doi: 10.1186/s12885-019-5380-3 (PMC6399859; doi:10.1186/s12885-019-5380-3)
Supplement: Supplementary file 1 — Table S1. Institutional Review Board or Ethics Committee of Each Participating Site. Description of data: Provides details of institutional review board/ethics committees for each participating site. (PDF 118 kb) [file 12885_2019_5380_MOESM1_ESM.pdf]

Supplemental Table: Institutional Review Board or Ethics Committee of Each Participating Site

Country	Site	PI	Site	IRB/EC Name
Canada	0016	Siu, Lillian	Princess Margaret Cancer Centre	University Health Network Research Ethics Board
France	0028	Soria, Jean-Charles (Andrea Varga)	Institut Gustave Roussy,	Comité de protection des personnes Ile-de-France III
Italy	0052	Braud, Filippo De	Fondazione IRCCS Istituto Nazionale Tumori,	Istituto Nazionale dei Tumori. Fondazione IRCSS, Comitato Etico Indipendente
Japan	0031	Tamura, Kenji	National Cancer Center Hospital Tokyo	Institutional Review Board for National Cancer Center
Japan	0032	Doi, Toshihiko	National Cancer Center Hospital East	Institutional Review Board for National Cancer Center
Korea, Republic of	0034	Bang, Yung-Jue	Seoul National University Hospital	Seoul National University Hospital, IRB Biomedical Research Institute
Taiwan	0037	Hsu, Chiun	National Taiwan University Hospital,	National Taiwan University Hospital, Ethics Review Committee
United States	0001	Cohen, Roger	Abramson Cancer Center at the University of Pennsylvania	University of Pennsylvania, Office of Regulatory Affairs

				Institutional Review Board
United States	0002	Borazanci, Erkut (Ramesh K. Ramanathan, MD/Samuel Ejadi)	AZ	Western Institutional Review Board
United States	0003	Piha-Paul, Sarina	The University of Texas MD Anderson Cancer Center	University of Texas MD Anderson Cancer Center Institute Review Board
United States	0007	Rugo, Hope	University of California, San Francisco	University of California, San Francisco Committee on Human Research
United States	0009	Mehnert, Janice (Antoinette Tan, MD)	Rutgers Cancer Institute of New Jersey	Western Institutional Review Board
United States	0010	Ancell, Kristin (Igor Puzanov/Aimee Agnel)	Vanderbilt University School of Medicine	Vanderbilt University Institutional Review Board
